# *N*-Ethyl-2-[1-(2-hy­droxy-4-methyl­phen­yl)ethyl­idene]hydrazinecarbo­thio­amide

**DOI:** 10.1107/S1600536814012203

**Published:** 2014-05-31

**Authors:** Brian J. Anderson, Jeffrey R. Hall, Jerry P. Jasinski

**Affiliations:** a Department of Chemistry, Keene State College, 229 Main Street, Keene, NH 03435-2001, USA

## Abstract

The title compound, C_12_H_17_N_3_OS, crystallizes with two independent mol­ecules (*A* and *B*) in the asymmetric unit. The dihedral angle between the mean planes of the benzene ring and the hydrazinecarbo­thio­amide group are 6.9 (4) and 37.2 (5)° in mol­ecules *A* and *B*, respectively. An intra­molecular O—H⋯N hydrogen bond is observed in each mol­ecule. This serves to maintain an approximately planar conformation for mol­ecule *A*, but leaves a significant twist between these two groups in mol­ecule *B*. In the crystal, a weak N—H⋯S inter­action is observed, forming inversion dimers among the *B* mol­ecules and resulting in an *R*_2_^2^(8) motif. These dimers are further inter­connected by weak N—H⋯O and C—H⋯O inter­molecular inter­actions, forming chains along [011].

## Related literature   

For the biological activity of thio­semicarbazones, see: Chellan *et al.* (2010 ▶). For binding motifs of thio­semicarbazones, see: Lobana *et al.* (2009 ▶). For thio­semicarbazones as ligands in catalysis, see: Xie *et al.* (2010 ▶). For related structures, see: Anderson *et al.* (2012 ▶, 2013*a* ▶,*b* ▶).
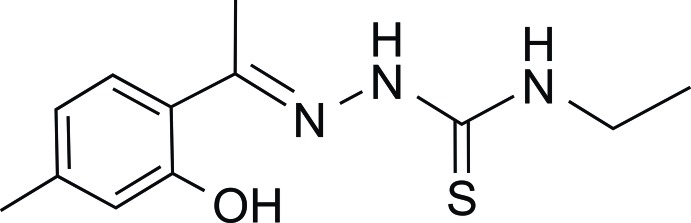


## Experimental   

### 

#### Crystal data  

C_12_H_17_N_3_OS
*M* *_r_* = 251.34
Triclinic, 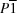

*a* = 7.4253 (4) Å
*b* = 8.7713 (4) Å
*c* = 20.7093 (11) Å
α = 96.238 (4)°
β = 94.400 (5)°
γ = 100.177 (4)°
*V* = 1313.35 (12) Å^3^
*Z* = 4
Mo *K*α radiation
μ = 0.24 mm^−1^
*T* = 173 K
0.28 × 0.24 × 0.12 mm

#### Data collection  

Agilent Eos Gemini diffractometer
Absorption correction: multi-scan (*CrysAlis PRO* and *CrysAlis RED*; Agilent, 2012 ▶) *T*_min_ = 0.693, *T*_max_ = 1.000
17011 measured reflections
8692 independent reflections
5875 reflections with *I* > 2σ(*I*)
*R*_int_ = 0.038

#### Refinement  

*R*[*F* ^2^ > 2σ(*F* ^2^)] = 0.070
*wR*(*F* ^2^) = 0.204
*S* = 1.09
8692 reflections
315 parameters
H-atom parameters constrained
Δρ_max_ = 0.66 e Å^−3^
Δρ_min_ = −0.38 e Å^−3^

### 

Data collection: *CrysAlis PRO* (Agilent, 2012 ▶); cell refinement: *CrysAlis PRO*; data reduction: *CrysAlis RED* (Agilent, 2012 ▶); program(s) used to solve structure: *SUPERFLIP* (Palatinus & Chapuis, 2007 ▶; Palatinus & van der Lee, 2008 ▶; Palatinus *et al.*, 2012 ▶).; program(s) used to refine structure: *SHELXL2012* (Sheldrick, 2008 ▶); molecular graphics: *OLEX2* (Dolomanov *et al.*, 2009 ▶); software used to prepare material for publication: *OLEX2*.

## Supplementary Material

Crystal structure: contains datablock(s) I. DOI: 10.1107/S1600536814012203/fj2676sup1.cif

Structure factors: contains datablock(s) I. DOI: 10.1107/S1600536814012203/fj2676Isup2.hkl

Supporting information file. DOI: 10.1107/S1600536814012203/fj2676Isup3.cml

CCDC reference: 1005355

Additional supporting information:  crystallographic information; 3D view; checkCIF report

## Figures and Tables

**Table 1:** Hydrogen-bond geometry (Å, °)

*D*—H⋯*A*	*D*—H	H⋯*A*	*D*⋯*A*	*D*—H⋯*A*
O1*A*—H1*A*⋯N3*A*	0.84	1.85	2.589 (2)	146
C10*A*—H10*B*⋯O1*B* ^i^	0.98	2.45	3.406 (3)	164
O1*B*—H1*B*⋯N3*B*	0.84	1.81	2.545 (2)	146
N1*B*—H1*BA*⋯O1*A* ^ii^	0.88	2.36	3.076 (2)	139
N2*B*—H2*B*⋯S1*B* ^iii^	0.88	2.52	3.320 (2)	152

Symmetry codes: (i) 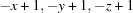; (ii) 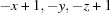; (iii) 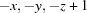.

**Fig. 1.**
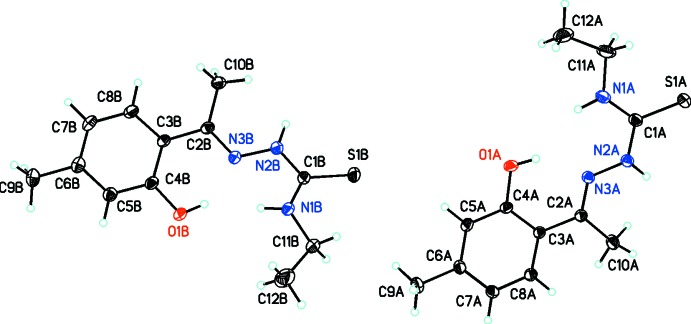
ORTEP drawing of (I), C12H17N3OS, showing the labeling scheme of molecules A and B with 30% probability displacement ellipsoids.

**Fig. 2.**
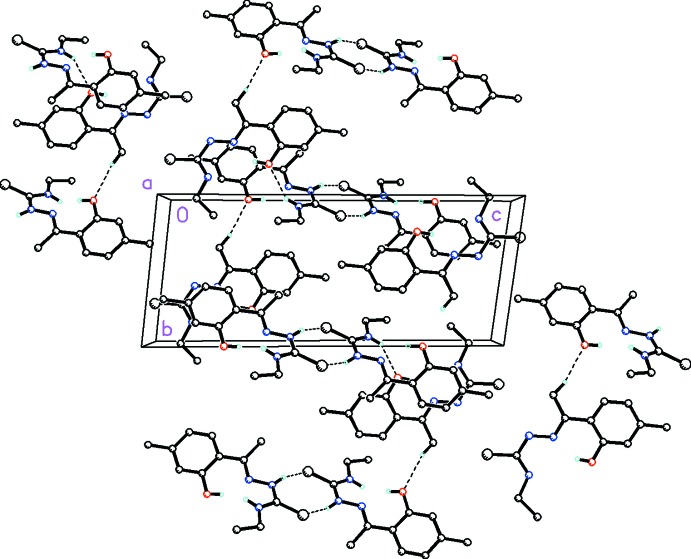
Molecular packing for (I) viewed along the a axis. Dashed lines indicate weak N2B—H2B···S1B intermolecular interactions forming inversion dimers among the B molecules in an R22[8] motif format. These dimers are further interconnected by weak N1B—H1BA···O1A and C10A—HH10B···O1B intermolecular interactions forming polymeric chains along [011].

**Fig. 3.**
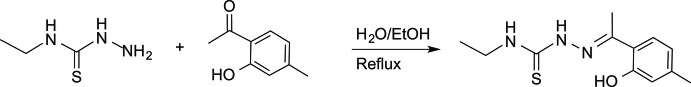
Reaction scheme.

## supplementary crystallographic information

### 1. Comment

Thiosemicarbazones are a versatile class of ligands that have been studied for their biological activity (Chellan *et al.*, 2010), interesting binding motifs (Lobana *et al.*, 2009), and their use as ligands in catalysis (Xie *et al.*, 2010). We have previously reported the structure of three similar novel thiosemicarbazones (Anderson *et al.*, 2012; Anderson *et al.*, 2013*a*; Anderson *et al.*, 2013*b*). Here, we report the synthesis and crystal structure of a new novel thiosemicarbazone ligand, (I), C_12_H_17_N_3_OS.

The title compound, (I), crystallizes with two independent molecules (A & B) in the asymmetric unit (Fig. 1). The dihedral angles between the mean planes of the benzene ring and the hydrazinecarbothioamide group is 6.9 (4)° (N3A/N2A/C1A/S1A/N1A) and 37.2 (5)° (N3B/N2B/C1B/S1B/N1B). An intramolecular O—H···N hydrogen bond is observed serving to maintain an approximately planar conformation in A. However in B there is a significant twist between these two groups. In the crystal, a weak N2B—H2B···S1B intermolecular interaction is observed forming inversion dimers among the B molecules in an R_2_^2^[8] motif format (Fig. 2). These dimers are further interconnected by weak N1B—H1BA···O1A and C10A—HH10B···O1B intermolecular interactions (Table 1) forming polymeric chains along [011].

### 2. Experimental

A 25 mL round bottom flask was charged with 0.1986 g (1.428 mmol) of 4'-methylacetophenone, 0.1702 g (1.428 mmol) of 4-ethyl-3-thiosemicarbazide and dissolved in 5 mL of a 1:1 ethanol: water solution and refluxed for 96 hours (Fig. 3). The reaction was allowed to cool to room temperature before dichloromethane (5 mL) and deionized water (5mL) were added, and the organic layer was separated. The aqueous layer was then extracted with an additional 5 mL of dichloromethane. The organic layers were then combined, washed with brine (2 X 5 mL), dried with magnesium sulfate, and the solvent removed in vacuo resulting in an off-white powder. The product was recrystallized from dichloromethane. m.p. 428–431 K.

### 3. Refinement

All of the H atoms were placed in their calculated positions and then refined using the riding model with Atom—H lengths of 0.95Å (CH), 0.99Å (CH_2_), 0.98Å (CH_3_), 0.88Å (NH) or 0.84Å (OH). Isotropic displacement parameters for these atoms were set to 1.2 (CH, CH_2_, NH) or 1.5 (CH_3_, OH) times *U*_eq_ of the parent atom. Idealised Me refined as rotating group. Idealised tetrahedral OH refined as rotating group.

### Crystal data

**Table:**

C_12_H_17_N_3_OS	*Z* = 4
*M**_r_* = 251.34	*F*(000) = 536
Triclinic, *P*1	*D*_x_ = 1.271 Mg m^−^^3^
*a* = 7.4253 (4) Å	Mo *K*α radiation, λ = 0.71073 Å
*b* = 8.7713 (4) Å	Cell parameters from 4160 reflections
*c* = 20.7093 (11) Å	θ = 3.6–32.3°
α = 96.238 (4)°	µ = 0.24 mm^−^^1^
β = 94.400 (5)°	*T* = 173 K
γ = 100.177 (4)°	Irregular, colourless
*V* = 1313.35 (12) Å^3^	0.28 × 0.24 × 0.12 mm

### Data collection

**Table:**

Agilent Eos Gemini diffractometer	8692 independent reflections
Radiation source: Enhance (Mo) X-ray Source	5875 reflections with *I* > 2σ(*I*)
Graphite monochromator	*R*_int_ = 0.038
Detector resolution: 16.0416 pixels mm^-1^	θ_max_ = 32.9°, θ_min_ = 3.1°
ω scans	*h* = −11→11
Absorption correction: multi-scan (*CrysAlis PRO* and *CrysAlis RED*; Agilent, 2012)	*k* = −12→13
*T*_min_ = 0.693, *T*_max_ = 1.000	*l* = −26→30
17011 measured reflections	

### Refinement

**Table:**

Refinement on *F*^2^	Primary atom site location: structure-invariant direct methods
Least-squares matrix: full	Hydrogen site location: inferred from neighbouring sites
*R*[*F*^2^ > 2σ(*F*^2^)] = 0.070	H-atom parameters constrained
*wR*(*F*^2^) = 0.204	*w* = 1/[σ^2^(*F*_o_^2^) + (0.0861*P*)^2^ + 0.5803*P*] where *P* = (*F*_o_^2^ + 2*F*_c_^2^)/3
*S* = 1.09	(Δ/σ)_max_ = 0.002
8692 reflections	Δρ_max_ = 0.66 e Å^−^^3^
315 parameters	Δρ_min_ = −0.38 e Å^−^^3^
0 restraints	

### Special details

**Table:**

Geometry. All esds (except the esd in the dihedral angle between two l.s. planes) are estimated using the full covariance matrix. The cell esds are taken into account individually in the estimation of esds in distances, angles and torsion angles; correlations between esds in cell parameters are only used when they are defined by crystal symmetry. An approximate (isotropic) treatment of cell esds is used for estimating esds involving l.s. planes.

### Fractional atomic coordinates and isotropic or equivalent isotropic displacement parameters (Å^2^)

**Table:**

	*x*	*y*	*z*	*U*_iso_*/*U*_eq_	
S1A	0.83372 (9)	0.27257 (8)	1.00813 (3)	0.04037 (17)	
O1A	0.6515 (3)	0.23324 (18)	0.72182 (8)	0.0367 (4)	
H1A	0.6753	0.2509	0.7625	0.055*	
N1A	0.6804 (4)	0.1452 (2)	0.89037 (10)	0.0445 (5)	
H1AA	0.6540	0.1539	0.8489	0.053*	
N2A	0.8328 (3)	0.3978 (2)	0.89858 (9)	0.0292 (4)	
H2A	0.8879	0.4857	0.9220	0.035*	
N3A	0.8001 (3)	0.3907 (2)	0.83175 (8)	0.0264 (3)	
C1A	0.7795 (3)	0.2685 (3)	0.92817 (10)	0.0308 (4)	
C2A	0.8520 (3)	0.5138 (2)	0.80405 (10)	0.0262 (4)	
C3A	0.8189 (3)	0.4995 (2)	0.73234 (10)	0.0244 (4)	
C4A	0.7229 (3)	0.3614 (2)	0.69440 (10)	0.0253 (4)	
C5A	0.6966 (3)	0.3517 (2)	0.62696 (10)	0.0288 (4)	
H5A	0.6272	0.2590	0.6027	0.035*	
C6A	0.7694 (3)	0.4746 (3)	0.59403 (10)	0.0295 (4)	
C7A	0.8656 (3)	0.6109 (3)	0.63058 (11)	0.0332 (5)	
H7A	0.9159	0.6967	0.6091	0.040*	
C8A	0.8889 (3)	0.6230 (2)	0.69794 (11)	0.0307 (4)	
H8A	0.9542	0.7179	0.7218	0.037*	
C9A	0.7471 (4)	0.4560 (3)	0.52077 (11)	0.0431 (6)	
H9AA	0.8343	0.3935	0.5038	0.065*	
H9AB	0.7712	0.5591	0.5056	0.065*	
H9AC	0.6213	0.4033	0.5050	0.065*	
C10A	0.9427 (5)	0.6663 (3)	0.84219 (12)	0.0476 (7)	
H10A	0.8864	0.6808	0.8832	0.071*	
H10B	0.9271	0.7515	0.8166	0.071*	
H10C	1.0741	0.6666	0.8518	0.071*	
C11A	0.6132 (6)	−0.0035 (3)	0.91371 (15)	0.0679 (11)	
H11A	0.5281	0.0117	0.9474	0.081*	
H11B	0.7177	−0.0439	0.9338	0.081*	
C12A	0.5152 (5)	−0.1188 (3)	0.85797 (16)	0.0609 (9)	
H12A	0.6030	−0.1424	0.8271	0.091*	
H12B	0.4188	−0.0745	0.8358	0.091*	
H12C	0.4596	−0.2149	0.8745	0.091*	
S1B	0.27526 (9)	0.11629 (8)	0.50079 (3)	0.03846 (17)	
O1B	0.0388 (3)	−0.00478 (19)	0.22569 (9)	0.0442 (5)	
H1B	0.0632	−0.0032	0.2661	0.066*	
N1B	0.3440 (3)	0.0524 (2)	0.37797 (9)	0.0328 (4)	
H1BA	0.3114	0.0004	0.3388	0.039*	
N2B	0.0643 (3)	−0.0663 (2)	0.40373 (9)	0.0322 (4)	
H2B	−0.0052	−0.1076	0.4323	0.039*	
N3B	0.0112 (3)	−0.0985 (2)	0.33722 (9)	0.0297 (4)	
C1B	0.2276 (3)	0.0313 (2)	0.42314 (11)	0.0302 (4)	
C2B	−0.1110 (3)	−0.2211 (2)	0.31448 (11)	0.0275 (4)	
C3B	−0.1631 (3)	−0.2420 (2)	0.24375 (10)	0.0269 (4)	
C4B	−0.0889 (3)	−0.1338 (2)	0.20249 (11)	0.0304 (4)	
C5B	−0.1424 (3)	−0.1555 (3)	0.13613 (12)	0.0335 (5)	
H5B	−0.0930	−0.0789	0.1099	0.040*	
C6B	−0.2666 (3)	−0.2867 (3)	0.10702 (12)	0.0341 (5)	
C7B	−0.3400 (3)	−0.3951 (3)	0.14698 (13)	0.0383 (5)	
H7B	−0.4254	−0.4858	0.1282	0.046*	
C8B	−0.2904 (3)	−0.3726 (3)	0.21335 (12)	0.0350 (5)	
H8B	−0.3441	−0.4479	0.2394	0.042*	
C9B	−0.3194 (4)	−0.3111 (3)	0.03475 (12)	0.0442 (6)	
H9BA	−0.4496	−0.3057	0.0261	0.066*	
H9BB	−0.2987	−0.4138	0.0166	0.066*	
H9BC	−0.2443	−0.2298	0.0143	0.066*	
C10B	−0.1944 (3)	−0.3386 (3)	0.35613 (12)	0.0354 (5)	
H10D	−0.3220	−0.3281	0.3611	0.053*	
H10E	−0.1241	−0.3208	0.3992	0.053*	
H10F	−0.1918	−0.4440	0.3354	0.053*	
C11B	0.5237 (4)	0.1568 (3)	0.38931 (12)	0.0419 (6)	
H11C	0.6026	0.1200	0.4227	0.050*	
H11D	0.5078	0.2634	0.4058	0.050*	
C12B	0.6130 (6)	0.1606 (6)	0.32810 (19)	0.0867 (14)	
H12D	0.5308	0.1899	0.2942	0.130*	
H12E	0.6394	0.0572	0.3141	0.130*	
H12F	0.7281	0.2373	0.3354	0.130*	

### Atomic displacement parameters (Å^2^)

**Table:**

	*U*^11^	*U*^22^	*U*^33^	*U*^12^	*U*^13^	*U*^23^
S1A	0.0474 (4)	0.0438 (3)	0.0272 (3)	−0.0004 (3)	0.0005 (2)	0.0092 (2)
O1A	0.0507 (10)	0.0243 (7)	0.0290 (8)	−0.0091 (7)	0.0076 (7)	−0.0001 (6)
N1A	0.0690 (15)	0.0318 (10)	0.0271 (10)	−0.0068 (10)	0.0037 (9)	0.0063 (8)
N2A	0.0386 (10)	0.0245 (8)	0.0234 (8)	0.0036 (7)	0.0032 (7)	0.0019 (6)
N3A	0.0328 (9)	0.0235 (8)	0.0227 (8)	0.0047 (7)	0.0042 (6)	0.0019 (6)
C1A	0.0380 (11)	0.0282 (10)	0.0264 (10)	0.0050 (9)	0.0067 (8)	0.0042 (8)
C2A	0.0319 (10)	0.0189 (8)	0.0275 (10)	0.0053 (8)	−0.0001 (8)	0.0018 (7)
C3A	0.0279 (9)	0.0185 (8)	0.0256 (9)	0.0031 (7)	0.0001 (7)	0.0013 (7)
C4A	0.0266 (9)	0.0202 (8)	0.0280 (10)	0.0015 (7)	0.0041 (7)	0.0018 (7)
C5A	0.0297 (10)	0.0253 (9)	0.0286 (10)	0.0019 (8)	0.0005 (8)	−0.0024 (7)
C6A	0.0342 (10)	0.0287 (10)	0.0254 (10)	0.0069 (9)	−0.0004 (8)	0.0025 (7)
C7A	0.0439 (12)	0.0249 (9)	0.0294 (11)	0.0014 (9)	−0.0008 (9)	0.0075 (8)
C8A	0.0395 (11)	0.0195 (8)	0.0307 (11)	0.0017 (8)	−0.0022 (8)	0.0030 (7)
C9A	0.0560 (16)	0.0437 (13)	0.0259 (11)	0.0024 (12)	−0.0011 (10)	0.0030 (9)
C10A	0.080 (2)	0.0258 (11)	0.0280 (12)	−0.0066 (12)	−0.0112 (12)	0.0026 (8)
C11A	0.120 (3)	0.0359 (14)	0.0379 (15)	−0.0174 (17)	0.0104 (17)	0.0114 (11)
C12A	0.079 (2)	0.0331 (13)	0.062 (2)	−0.0119 (15)	0.0111 (16)	0.0024 (12)
S1B	0.0403 (3)	0.0435 (3)	0.0269 (3)	−0.0003 (3)	0.0034 (2)	−0.0028 (2)
O1B	0.0627 (12)	0.0257 (8)	0.0350 (9)	−0.0126 (8)	−0.0058 (8)	0.0044 (6)
N1B	0.0359 (9)	0.0302 (9)	0.0274 (9)	−0.0044 (8)	0.0045 (7)	−0.0021 (7)
N2B	0.0342 (9)	0.0326 (9)	0.0271 (9)	−0.0002 (8)	0.0056 (7)	0.0000 (7)
N3B	0.0313 (9)	0.0275 (8)	0.0281 (9)	0.0017 (7)	0.0014 (7)	0.0000 (7)
C1B	0.0345 (10)	0.0262 (9)	0.0280 (10)	0.0023 (9)	0.0004 (8)	0.0019 (8)
C2B	0.0271 (9)	0.0222 (9)	0.0329 (11)	0.0047 (8)	0.0058 (8)	0.0007 (7)
C3B	0.0263 (9)	0.0211 (9)	0.0325 (11)	0.0043 (8)	0.0027 (8)	0.0000 (7)
C4B	0.0345 (11)	0.0198 (9)	0.0351 (11)	0.0035 (8)	0.0003 (8)	0.0011 (8)
C5B	0.0361 (11)	0.0277 (10)	0.0364 (12)	0.0070 (9)	0.0002 (9)	0.0039 (8)
C6B	0.0327 (11)	0.0321 (11)	0.0370 (12)	0.0104 (9)	−0.0016 (9)	−0.0015 (9)
C7B	0.0328 (11)	0.0312 (11)	0.0442 (14)	−0.0034 (9)	−0.0032 (9)	−0.0046 (9)
C8B	0.0331 (11)	0.0274 (10)	0.0412 (13)	−0.0019 (9)	0.0038 (9)	0.0014 (9)
C9B	0.0433 (13)	0.0496 (15)	0.0361 (13)	0.0088 (12)	−0.0071 (10)	−0.0032 (11)
C10B	0.0385 (12)	0.0301 (11)	0.0359 (12)	−0.0005 (9)	0.0085 (9)	0.0042 (9)
C11B	0.0397 (12)	0.0426 (13)	0.0351 (13)	−0.0111 (11)	0.0015 (10)	0.0010 (10)
C12B	0.067 (2)	0.104 (3)	0.067 (2)	−0.037 (2)	0.0288 (18)	−0.016 (2)

### Geometric parameters (Å, º)

**Table:**

S1A—C1A	1.669 (2)	S1B—C1B	1.681 (2)
O1A—H1A	0.8400	O1B—H1B	0.8400
O1A—C4A	1.357 (2)	O1B—C4B	1.358 (3)
N1A—H1AA	0.8800	N1B—H1BA	0.8800
N1A—C1A	1.330 (3)	N1B—C1B	1.327 (3)
N1A—C11A	1.459 (3)	N1B—C11B	1.465 (3)
N2A—H2A	0.8800	N2B—H2B	0.8800
N2A—N3A	1.379 (2)	N2B—N3B	1.387 (3)
N2A—C1A	1.359 (3)	N2B—C1B	1.360 (3)
N3A—C2A	1.290 (3)	N3B—C2B	1.297 (3)
C2A—C3A	1.474 (3)	C2B—C3B	1.469 (3)
C2A—C10A	1.496 (3)	C2B—C10B	1.497 (3)
C3A—C4A	1.412 (3)	C3B—C4B	1.414 (3)
C3A—C8A	1.406 (3)	C3B—C8B	1.404 (3)
C4A—C5A	1.386 (3)	C4B—C5B	1.384 (3)
C5A—H5A	0.9500	C5B—H5B	0.9500
C5A—C6A	1.389 (3)	C5B—C6B	1.388 (3)
C6A—C7A	1.390 (3)	C6B—C7B	1.394 (3)
C6A—C9A	1.501 (3)	C6B—C9B	1.500 (3)
C7A—H7A	0.9500	C7B—H7B	0.9500
C7A—C8A	1.383 (3)	C7B—C8B	1.379 (3)
C8A—H8A	0.9500	C8B—H8B	0.9500
C9A—H9AA	0.9800	C9B—H9BA	0.9800
C9A—H9AB	0.9800	C9B—H9BB	0.9800
C9A—H9AC	0.9800	C9B—H9BC	0.9800
C10A—H10A	0.9800	C10B—H10D	0.9800
C10A—H10B	0.9800	C10B—H10E	0.9800
C10A—H10C	0.9800	C10B—H10F	0.9800
C11A—H11A	0.9900	C11B—H11C	0.9900
C11A—H11B	0.9900	C11B—H11D	0.9900
C11A—C12A	1.497 (4)	C11B—C12B	1.476 (4)
C12A—H12A	0.9800	C12B—H12D	0.9800
C12A—H12B	0.9800	C12B—H12E	0.9800
C12A—H12C	0.9800	C12B—H12F	0.9800
			
C4A—O1A—H1A	109.5	C4B—O1B—H1B	109.5
C1A—N1A—H1AA	118.1	C1B—N1B—H1BA	118.0
C1A—N1A—C11A	123.7 (2)	C1B—N1B—C11B	124.01 (19)
C11A—N1A—H1AA	118.1	C11B—N1B—H1BA	118.0
N3A—N2A—H2A	120.1	N3B—N2B—H2B	121.2
C1A—N2A—H2A	120.1	C1B—N2B—H2B	121.2
C1A—N2A—N3A	119.78 (17)	C1B—N2B—N3B	117.55 (18)
C2A—N3A—N2A	119.35 (17)	C2B—N3B—N2B	120.16 (18)
N1A—C1A—S1A	123.78 (17)	N1B—C1B—S1B	123.27 (17)
N1A—C1A—N2A	116.28 (19)	N1B—C1B—N2B	116.29 (19)
N2A—C1A—S1A	119.91 (16)	N2B—C1B—S1B	120.44 (17)
N3A—C2A—C3A	117.47 (17)	N3B—C2B—C3B	116.11 (18)
N3A—C2A—C10A	122.20 (19)	N3B—C2B—C10B	123.4 (2)
C3A—C2A—C10A	120.34 (18)	C3B—C2B—C10B	120.51 (18)
C4A—C3A—C2A	122.63 (17)	C4B—C3B—C2B	122.56 (18)
C8A—C3A—C2A	120.90 (17)	C8B—C3B—C2B	121.23 (19)
C8A—C3A—C4A	116.43 (18)	C8B—C3B—C4B	116.2 (2)
O1A—C4A—C3A	122.03 (18)	O1B—C4B—C3B	121.90 (19)
O1A—C4A—C5A	116.98 (17)	O1B—C4B—C5B	116.80 (19)
C5A—C4A—C3A	121.00 (18)	C5B—C4B—C3B	121.29 (19)
C4A—C5A—H5A	119.3	C4B—C5B—H5B	119.3
C4A—C5A—C6A	121.46 (19)	C4B—C5B—C6B	121.4 (2)
C6A—C5A—H5A	119.3	C6B—C5B—H5B	119.3
C5A—C6A—C7A	118.27 (19)	C5B—C6B—C7B	118.0 (2)
C5A—C6A—C9A	119.8 (2)	C5B—C6B—C9B	120.9 (2)
C7A—C6A—C9A	121.9 (2)	C7B—C6B—C9B	121.1 (2)
C6A—C7A—H7A	119.7	C6B—C7B—H7B	119.5
C8A—C7A—C6A	120.64 (19)	C8B—C7B—C6B	120.9 (2)
C8A—C7A—H7A	119.7	C8B—C7B—H7B	119.5
C3A—C8A—H8A	118.9	C3B—C8B—H8B	118.9
C7A—C8A—C3A	122.15 (19)	C7B—C8B—C3B	122.2 (2)
C7A—C8A—H8A	118.9	C7B—C8B—H8B	118.9
C6A—C9A—H9AA	109.5	C6B—C9B—H9BA	109.5
C6A—C9A—H9AB	109.5	C6B—C9B—H9BB	109.5
C6A—C9A—H9AC	109.5	C6B—C9B—H9BC	109.5
H9AA—C9A—H9AB	109.5	H9BA—C9B—H9BB	109.5
H9AA—C9A—H9AC	109.5	H9BA—C9B—H9BC	109.5
H9AB—C9A—H9AC	109.5	H9BB—C9B—H9BC	109.5
C2A—C10A—H10A	109.5	C2B—C10B—H10D	109.5
C2A—C10A—H10B	109.5	C2B—C10B—H10E	109.5
C2A—C10A—H10C	109.5	C2B—C10B—H10F	109.5
H10A—C10A—H10B	109.5	H10D—C10B—H10E	109.5
H10A—C10A—H10C	109.5	H10D—C10B—H10F	109.5
H10B—C10A—H10C	109.5	H10E—C10B—H10F	109.5
N1A—C11A—H11A	109.7	N1B—C11B—H11C	109.6
N1A—C11A—H11B	109.7	N1B—C11B—H11D	109.6
N1A—C11A—C12A	109.8 (2)	N1B—C11B—C12B	110.2 (2)
H11A—C11A—H11B	108.2	H11C—C11B—H11D	108.1
C12A—C11A—H11A	109.7	C12B—C11B—H11C	109.6
C12A—C11A—H11B	109.7	C12B—C11B—H11D	109.6
C11A—C12A—H12A	109.5	C11B—C12B—H12D	109.5
C11A—C12A—H12B	109.5	C11B—C12B—H12E	109.5
C11A—C12A—H12C	109.5	C11B—C12B—H12F	109.5
H12A—C12A—H12B	109.5	H12D—C12B—H12E	109.5
H12A—C12A—H12C	109.5	H12D—C12B—H12F	109.5
H12B—C12A—H12C	109.5	H12E—C12B—H12F	109.5
			
O1A—C4A—C5A—C6A	−177.9 (2)	O1B—C4B—C5B—C6B	177.4 (2)
N2A—N3A—C2A—C3A	178.30 (17)	N2B—N3B—C2B—C3B	177.79 (18)
N2A—N3A—C2A—C10A	−1.6 (3)	N2B—N3B—C2B—C10B	−3.3 (3)
N3A—N2A—C1A—S1A	175.07 (16)	N3B—N2B—C1B—S1B	162.66 (16)
N3A—N2A—C1A—N1A	−6.7 (3)	N3B—N2B—C1B—N1B	−18.2 (3)
N3A—C2A—C3A—C4A	4.4 (3)	N3B—C2B—C3B—C4B	−1.4 (3)
N3A—C2A—C3A—C8A	−173.1 (2)	N3B—C2B—C3B—C8B	178.3 (2)
C1A—N1A—C11A—C12A	−177.2 (3)	C1B—N1B—C11B—C12B	−176.4 (3)
C1A—N2A—N3A—C2A	−179.6 (2)	C1B—N2B—N3B—C2B	160.1 (2)
C2A—C3A—C4A—O1A	1.3 (3)	C2B—C3B—C4B—O1B	1.3 (3)
C2A—C3A—C4A—C5A	−179.14 (19)	C2B—C3B—C4B—C5B	−179.1 (2)
C2A—C3A—C8A—C7A	177.6 (2)	C2B—C3B—C8B—C7B	−179.4 (2)
C3A—C4A—C5A—C6A	2.6 (3)	C3B—C4B—C5B—C6B	−2.1 (3)
C4A—C3A—C8A—C7A	0.0 (3)	C4B—C3B—C8B—C7B	0.3 (3)
C4A—C5A—C6A—C7A	−2.0 (3)	C4B—C5B—C6B—C7B	1.5 (3)
C4A—C5A—C6A—C9A	176.5 (2)	C4B—C5B—C6B—C9B	−178.2 (2)
C5A—C6A—C7A—C8A	0.4 (4)	C5B—C6B—C7B—C8B	−0.1 (4)
C6A—C7A—C8A—C3A	0.6 (4)	C6B—C7B—C8B—C3B	−0.8 (4)
C8A—C3A—C4A—O1A	178.9 (2)	C8B—C3B—C4B—O1B	−178.4 (2)
C8A—C3A—C4A—C5A	−1.5 (3)	C8B—C3B—C4B—C5B	1.1 (3)
C9A—C6A—C7A—C8A	−178.0 (2)	C9B—C6B—C7B—C8B	179.7 (2)
C10A—C2A—C3A—C4A	−175.7 (2)	C10B—C2B—C3B—C4B	179.7 (2)
C10A—C2A—C3A—C8A	6.8 (3)	C10B—C2B—C3B—C8B	−0.6 (3)
C11A—N1A—C1A—S1A	−2.5 (4)	C11B—N1B—C1B—S1B	−2.9 (3)
C11A—N1A—C1A—N2A	179.4 (3)	C11B—N1B—C1B—N2B	178.0 (2)

### Hydrogen-bond geometry (Å, º)

**Table:**

*D*—H···*A*	*D*—H	H···*A*	*D*···*A*	*D*—H···*A*
O1*A*—H1*A*···N3*A*	0.84	1.85	2.589 (2)	146
C10*A*—H10*B*···O1*B*^i^	0.98	2.45	3.406 (3)	164
O1*B*—H1*B*···N3*B*	0.84	1.81	2.545 (2)	146
N1*B*—H1*BA*···O1*A*^ii^	0.88	2.36	3.076 (2)	139
N2*B*—H2*B*···S1*B*^iii^	0.88	2.52	3.320 (2)	152

Symmetry codes: (i) −*x*+1, −*y*+1, −*z*+1; (ii) −*x*+1, −*y*, −*z*+1; (iii) −*x*, −*y*, −*z*+1.
